# Effect of *ABCB1* C3435T polymorphism on docetaxel pharmacokinetics according to menopausal status in breast cancer patients

**DOI:** 10.1038/sj.bjc.6605789

**Published:** 2010-07-13

**Authors:** A Fajac, J Gligorov, K Rezai, P Lévy, E Lévy, F Selle, K Beerblock, D Avenin, P Saintigny, S Hugonin, J-F Bernaudin, F Lokiec

**Affiliations:** 1 Service d’Histologie-Biologie Tumorale, hôpital Tenon, AP-HP, ER2 UPMC Université Pierre et Marie Curie, 4 rue de la Chine, Paris 75020, France; 2 Service d’Oncologie, hôpital Tenon, AP-HP, 4 rue de la Chine, Paris 75020, France; 3 Service de Pharmacologie, Centre René Huguenin, 35 rue Dailly, St-Cloud 92210, France; 4 Département de Santé Publique, hôpital Tenon, AP-HP, Inserm UMR S 707, Université Pierre et Marie Curie, 4 rue de la Chine, Paris 75020, France; 5 Service d’Oncologie, HEGP, AP-HP, 20 rue Leblanc, Paris 75015, France; 6 Service d’Oncologie, Hôpital Avicenne, AP-HP, 125 rue Stalingrad, Bobigny 93000, France

**Keywords:** ABCB1, polymorphisms, docetaxel, pharmacokinetics, breast cancer

## Abstract

**Background:**

It can be hypothesised that inherited polymorphisms in the drug-transporter *ABCB1* gene may interfere with interindividual variations in drug response in breast cancer patients. Docetaxel is a substrate for ABCB1 whose function has been shown to be modulated by oestrogen and progesterone.

**Methods:**

Whether *ABCB1* polymorphisms including T-129C, A61G, C1236T, G2677T/A and C3435T polymorphisms could account for variations in the disposition of docetaxel and whether menopausal status at the time of diagnosis might interact with this effect were analysed in women receiving neoadjuvant chemotherapy for breast cancer (*n*=86).

**Results:**

A highly significant association was observed, but restricted to premenopausal women (*n*=53), between the pharmacokinetics of docetaxel and C3435T polymorphism, as patients with CC genotype had lower mean values of the area under the plasma concentration-time curve (AUC) of docetaxel than patients with CT and TT genotypes (*P*<0.0001). Comparison between pre- and postmenopausal women with the same C3435T genotype yielded a significant difference in docetaxel AUC only for CC genotype (*P*<0.0001).

**Conclusion:**

These results suggest that C3435T polymorphism genotyping and menopausal status at the time of diagnosis might be useful when considering chemotherapy regimens including docetaxel in breast cancer patients.

Taxanes, drugs frequently used to treat breast cancer, are substrates for the ATP-binding cassette (ABC) transporter ABCB1 (Scala *et al*, 1997). This protein was initially discovered as an efflux transporter involved in multidrug resistance of tumour cells (Gottesman *et al*, 2002). However, ABCB1 is also physiologically expressed at the apical surface of epithelial cells in various organs responsible for drug disposition such as the intestine, liver and kidney and at the apical surface of endothelial cells controlling the availability of drugs at the blood–tissue interface (Schinkel, 1997). ABCB1 is therefore also involved in drug metabolism by having a role in the so-called phase 0 (efflux of unmodified drug) of the metabolism of xenobiotics.

Breast cancer patients treated with taxanes such as docetaxel show major interindividual variations in drug response, which might be at least partly because of inherited polymorphisms in genes encoding proteins involved in drug efflux including ABCB1 transporter (Relling and Dervieux, 2001; Rodrigues *et al*, 2008). Since the first systematic screening for polymorphisms in *ABCB1* coding region and the report of a functional polymorphism (Hoffmeyer *et al*, 2000), >45 polymorphisms have been described in coding regions, the promoter and non-coding regions (Kroetz *et al*, 2003). The polymorphisms for which the frequency of the variant allele is >5% include the T-129C in the promoter region, the synonymous C1236T (exon 12) and C3435T (exon 26) polymorphisms and the non-synonymous A61G (exon 2) and G2677T/A (exon 21) polymorphisms.

There is evidence that the sex steroids oestrogen and progesterone interfere with ABCB1 function as either substrates and/or regulators of ABCB1 expression. Oestrogens such as oestrone and oestriol have been shown to be a substrate of ABCB1 and to increase ABCB1 protein levels (Kim and Benet, 2004; Evseenko *et al*, 2007). Moreover, although not a substrate of ABCB1, progesterone has been reported to inhibit ABCB1-mediated efflux (Yang *et al*, 1989; Barnes *et al*, 1996; Hamilton *et al*, 2001) and increase *ABCB1* mRNA (Piekarz *et al*, 1993) and ATPase activity (Barnes *et al*, 1996; Kim and Benet, 2004).

To evaluate whether variations in disposition of docetaxel because of *ABCB1* polymorphisms could be involved in the response variability of breast cancer patients, the pharmacokinetics of this drug and inherited polymorphisms of *ABCB1* gene including T-129C, A61G, C1236T, G2677T/A and C3435T polymorphisms were analysed. Given that the hallmark of menopause is a decrease in ovarian secretion of oestrogen and progesterone, we analysed the effects of menopausal status at the time of diagnosis on the relationships between *ABCB1* polymorphisms and the pharmacokinetics of docetaxel given in neoadjuvant chemotherapy regimens.

## Patients and Methods

### Patients

Women with breast cancer receiving neoadjuvant chemotherapy were included in this study (*n*=86), namely patients with tumours larger than 20 mm (T2, T3 and T4 tumours) and absence of metastatic disease. Patient characteristics are listed in Table 1. A majority of the patients were Caucasians (*n*=66). Menopausal status at the time of diagnosis was specified. Menopause was defined as amenorrhea for at least 12 months. Mean age at diagnosis was 41 years (range: 26–56) for premenopausal patients and 61 years (range: 50–76) for postmenopausal patients.

Chemotherapy consisted of four cycles of doxorubicin (60 mg m^–2^) and cyclophosphamide (600 mg m^–2^) followed by four cycles of docetaxel (100 mg m^–2^). Trastuzumab was administered to four patients during courses of docetaxel because of the presence of c*erbB2* amplification in tumour cells.

The protocol was approved by the independent ethics committee of Pitié-Salpêtrière Hospital, Paris, France and all patients provided written informed consent before inclusion in the study including specific written informed consent for the pharmacogenetic analysis.

### Pharmacokinetics of docetaxel

Pharmacokinetic analysis was performed for the first course of docetaxel. A limited sampling strategy was used according to previously reported studies (Baille *et al*, 1997). Five heparinised blood samples (5 ml each) were required: immediately before infusion, 5 min before the end of infusion and 20 min, 2 h and 5 h after the end of infusion. After immediate centrifugation of the blood samples, plasma was stored at −20°C until further analysis. Plasma concentrations of docetaxel were determined using validated high-performance liquid chromatography methods with UV detection (Vergniol *et al*, 1992). The analytical range for docetaxel determination was 25–5000 ng ml^–1^. Individual drug clearances were estimated from docetaxel population pharmacokinetic parameters (Bruno *et al*, 1998) using the *POST HOC* option of NONMEM (Beal and Sheiner, 1998). The area under the plasma concentration-time curve (AUC) was calculated as AUC = dose/clearance.

### Genotype

Genomic DNA was extracted from whole blood (10 ml) using QIAamp DNA blood Maxi Kit (Qiagen, Hilden, Germany). T-129C (rs3213619), A61G (rs9282564), C1236T (rs1128503) and C3435T (rs1045642) polymorphisms were each analysed using two matching primers and two TaqMan MGB probes labeled with 6-FAM or VIC dye for allelic discrimination (assay IDs: C__27487486_10 and C__7586657_20 for T-129C and C3435T, respectively and custom-designed assays for A61G and C1236T with probes FAM (5′-AACTGAACGATAAAAG-3′) and VIC (5′-TTTAAACTGAACAATAAAAG-3′) and FAM (5′-TCAGGTTCAGGCCCTT-3′) and VIC (5′-TCAGGTTCAGACCCTT-3′), respectively, Applied Biosystems, Courtaboeuf, France). DNA (2.5 *μ*l) was amplified with TaqMan PCR Universal Master Mix (Applied Biosystems) and Assay Mix (Applied Biosystems) in a final volume of 25 *μ*l. Forty cycles with denaturation at 92°C, and annealing and extension at 60°C were performed.

For G2677T/A (rs2032582) polymorphism, the analysis was based on PCR-RFLP. The primer sequences designed by Primer3 software were 5′-TGACAAACGTTAGGCTTAAATTACA-3′ and 5′-AAGATTGCTTTGAGGAATGGTT-3′ for 2677F and 2677R, respectively. DNA (2.5 *μ*l) was amplified with 1.25 U Ampli Taq Gold DNA polymerase (Applied Biosystems), 200 nM 2677F and 2677R primers (Proligo, Paris, France), 200 *μ*M dNTP, 4 mM MgCl2 and GeneAmp Gold Buffer (Applied Biosystems) in a final volume of 25 *μ*l. Forty cycles with denaturation at 95°C, annealing at 63°C and extension at 72°C were performed. The 726 bp PCR product (12.5 *μ*l) was submitted to 2.5 U Bse YI (New England Biolabs Ozyme, Saint-Quentin en Yvelines, France) for 2 h at 37°C or 2.5 U Rsa I (New England Biolabs Ozyme) for 3 h at 37°C. Bse YI cuts at the G nucleotide yielding two fragments (590 and 136 kb). Rsa I enzyme cuts at the nonspecific 509 nucleotide and at the A variant yielding two fragments (508 and 218 kb) if A variant is absent, four fragments (508, 218, 136 and 82 kb) if one A allele is present and three fragments (508, 136 and 82 kb) if two A alleles are present. Digested PCR products were submitted to electrophoresis in Novex 8% TBE gels (Invitrogen, Eragny sur Oise, France).

### Statistical analysis

Mean values of AUC and mean values of clearance of docetaxel were compared between groups using the nonparametric Mann–Whitney test for two groups or the nonparametric Kruskal–Wallis test for three or more groups (StatView software, version 5.0; SAS Institute, Cary, NC, USA). Owing to multiple comparisons, statistical significance was defined as *P*<0.005.

## Results

Plasma concentrations of docetaxel were determined in the 86 patients. Genotyping results for the T-129C, A61G, C1236T, G2677T/A and C3435T polymorphisms, the two most frequent haplotypes for A61G, C1236T, G2677T/A and C3435T polymorphisms and the haplotype containing the homozygous variant genotypes, which have a frequency ⩾10%, are shown in Table 2.

For each polymorphism, the mean AUC of docetaxel was initially compared between each genotype using the nonparametric Mann–Whitney test for two groups or the nonparametric Kruskal–Wallis test for three or more groups. When a significant relationship was found for a polymorphism, pairwise comparisons were then performed between one genotype and the other genotypes for this polymorphism considered as a single group. The same analysis was performed for comparisons of mean clearances between genotypes for each polymorphism. Similar results were obtained whether AUC or clearances were considered as pharmacokinetic parameters for docetaxel. However, there was not always a strict correlation between AUC and clearance for a given genotype. This is probably because of the fact that AUC depends on docetaxel dose, which depends on body surface. The results below will be focused on AUC (for details on clearance, see the corresponding Tables).

**Figure 1:**
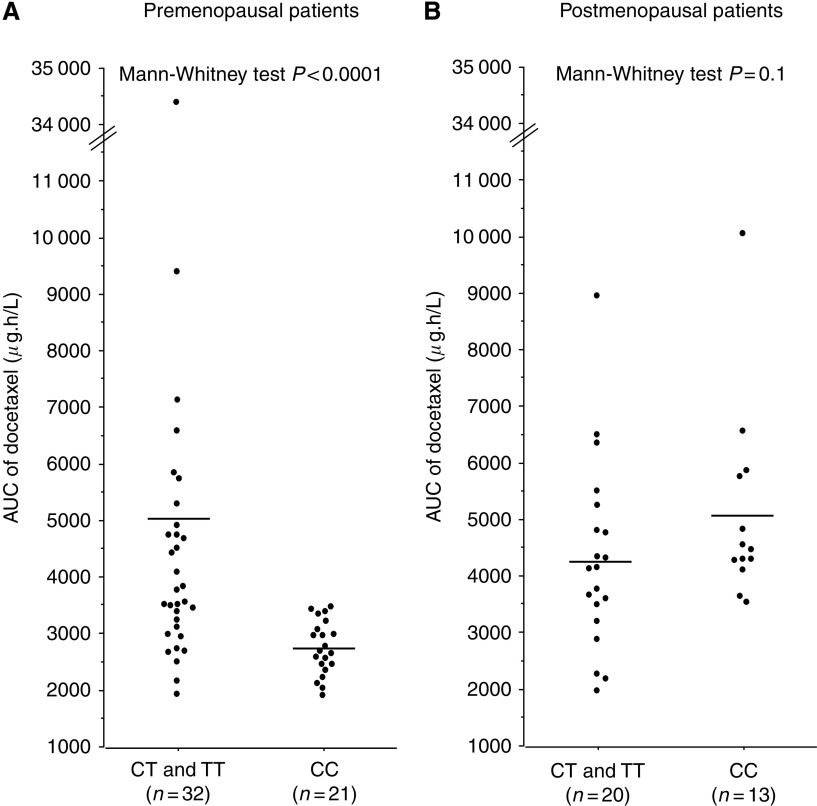
The aera under the plasma concentration-time curve (AUC) of docetaxel according to C3435T polymorphism and menopausal status. In (**A**) exclusion of the outlier patient with an AUC >34 000 *μ*g h l^–1^ did not alter the *P-*value. (**A**) Premenopausal patients. (**B**) Postmenopausal patients.

A striking association was observed between the pharmacokinetics of docetaxel and menopausal status at diagnosis. Mean values (± standard errors, s.e.) for AUC of docetaxel were lower in premenopausal women (4124±612 *μ*g h l^–1^, *n*=53) than in postmenopausal women (4598±298 *μ*g h l^–1^, *n*=33, Mann–Whitney test *P*=0.0008) (Figure 1).

The influence of menopausal status on the relationship between *ABCB1* polymorphisms and the pharmacokinetics of docetaxel was analysed. Results regarding the pharmacokinetics of docetaxel according to *ABCB1* polymorphisms in pre- and postmenopausal patients are summarised in Tables 3 and 4, respectively.

A significant association between C3435T polymorphism and the pharmacokinetics of docetaxel was observed in premenopausal women (Kruskal–Wallis test *P*=0.0002 for AUC, Table 3). The AUC of docetaxel was lower for 3435CC patients than for 3435CT and 3435TT patients considered as a single group (Mann-Whitney test *P*<0.0001, Figure 1A). In postmenopausal women, no significant relationship was found between the pharmacokinetics of docetaxel and C3435T polymorphism (Figure 1B, Table 4).

Analysis of the most frequent ethnic group in the study population, that is, Caucasians, showed that the relationship between 3435CC genotype and lower AUC remained significant in premenopausal women (AUC ± s.e. values (*μ*g h l^–1^): 2816±149 and 5094±1050 for 3435CC (*n*=10) *vs* 3435CT and 3435TT (*n*=30), respectively, Mann–Whitney test *P*=0.004).

Comparison between pre- and postmenopausal women with the same C3435T genotype yielded a significant difference in docetaxel AUC for CC genotype (Mann–Whitney test *P*<0.0001) with lower AUC in premenopausal women (see Tables 3 and 4: mean AUC (s.e.)=2727 *μ*g h l^–1^ (104) *vs* 5075 *μ*g h l^–1^ (481) for pre- *vs* postmenopausal 3435CC women, respectively). In contrast, no difference in docetaxel AUC was observed between pre- and postmenopausal women with CT genotype (Mann–Whitney test *P*=0.8) and between pre- and postmenopausal women with TT genotype (Mann–Whitney test *P*=0.4) (see Tables 3 and 4: mean AUC (s.e.)=5431 *μ*g h l^–1^ (1248) and 4279 *μ*g h l^–1^ (492) for pre- *vs* postmenopausal 3435CT women, respectively, and 3647 *μ*g h l^–1^ (455) and 4309 *μ*g h l^–1^ (539) for pre- *vs* postmenopausal 3435TT women, respectively).

No significant association (*P*<0.005) was found between the other polymorphisms analysed and docetaxel pharmacokinetics in premenopausal women (Table 3) or in postmenopausal women (Table 4). The 2677GG-3435CC diplotype and the most frequent 61AA-1236CC-2677GG-3435CC haplotype were significantly associated with lower AUC of docetaxel in premenopausal women (Table 3).

Analysis of the overall population did not show any significant relationship (*P*<0.005) between the pharmacokinetics of docetaxel and any of the *ABCB1* polymorphisms or any combination of *ABCB1* genotypes (Table 5).

## Discussion

This study shows an effect of menopausal status at diagnosis on the relationship between C3435T polymorphism of *ABCB*1 gene and the pharmacokinetics of docetaxel in breast cancer patients.

When evaluating the overall population, no association was observed between C3435T polymorphism and the pharmacokinetics of docetaxel, which is in accordance with the few published studies on this topic (Goh *et al*, 2002; Bosch *et al*, 2006; Tran *et al*, 2006; Lewis *et al*, 2007). In contrast, when analysing the population according to menopausal status at time of diagnosis of breast cancer, a highly significant association with docetaxel pharmacokinetics was observed in premenopausal women for this polymorphism (*P*<0.0001), but not in postmenopausal women. Comparison of pre- and postmenopausal women with the same C3435T genotype yielded a significant difference in docetaxel AUC only for the CC genotype (*P*<0.0001) and not for the CT or TT genotypes. Premenopausal CC women had significantly lower AUC than premenopausal (CT and TT) women and CC postmenopausal women. Premenopausal (CT and TT) women had similar AUC to those of postmenopausal CC and postmenopausal (CT and TT) women (see Figure 1). To the best of our knowledge, this is the first report of a specific effect of menopausal status at the time of diagnosis on the role of *ABCB1* polymorphisms in docetaxel disposition. By contrast, such an effect was not observed for doxorubicin in these patients (data not shown), which suggests that the effect is drug specific. A significant relationship (*P*=0.001) between docetaxel pharmacokinetics and C3435T genotype was also found for patients younger than 49 years, which was the mean age at diagnosis (data not shown). As this age is also the mean value of age at menopause, we believe that menopausal status at diagnosis rather than age has an effect on docetaxel pharmacokinetics.

The finding of a highly significant association in premenopausal women between lower AUC of docetaxel and 2677GG-3435CC diplotype and 61AA-1236CC-2677GG-3435CC haplotype is probably because of the strong linkage between these genotypes (Kroetz *et al*, 2003), although this remains to be evaluated in a large series of women receiving docetaxel neoadjuvant chemotherapy.

The 3435CC genotype has usually been associated with higher levels of ABCB1 mRNA and protein and increased drug efflux in normal tissues and tumours (Hoffmeyer *et al*, 2000; Hitzl *et al*, 2001; Tanabe *et al*, 2001; Fellay *et al*, 2002; Vaclavikova *et al*, 2008), although some discrepancies have been reported, especially in Japanese populations (Nakamura *et al*, 2002). It therefore makes sense to observe lower AUC of docetaxel in CC patients, as their higher levels of ABCB1 in organs involved in drug metabolism would result in higher efflux of docetaxel, higher elimination of the drug and subsequently lower AUC. The mechanisms by which this synonymous polymorphism affects ABCB1 function might be a lower mRNA stability of the 3435T variant (Wang *et al*, 2005) and/or a change in substrate binding site conformation of the variant ABCB1 protein (Kimchi-Sarfaty *et al*, 2007). This different protein folding could be due to a different rate of translation when rare codons are used which might be at least partly because of ribosome stalling (Fung and Gottesman, 2009).

Why lower AUC of docetaxel were specifically observed in premenopausal CC women and not in postmenopausal CC women is still unknown. A role for oestrogen and progesterone in modulating ABCB1 expression and function has been shown in various cells with increased ABCB1 expression induced by oestrogen and progesterone at both the mRNA and protein levels (Arceci *et al*, 1990; Kim and Benet, 2004; Evseenko *et al*, 2007). It may be hypothesised that this inductive action of oestrogen and progesterone on ABCB1 could at least partly explain the lower AUC of docetaxel observed in premenopausal women compared with postmenopausal women independently of any effect of C3435T polymorphism. Higher oestrogen and progesterone levels in premenopausal women would induce higher ABCB1 levels and consequently higher efflux of docetaxel. It can be hypothesised that the finding of lower docetaxel AUC only in premenopausal CC women might be due to an additive effect of genotype and hormones on ABCB1 overexpression. However, the hypothesised role of oestrogen and progesterone on docetaxel pharmacokinetics through ABCB1 expression must be interpreted in the light that these women had received previous chemotherapy inducing a possible decrease in oestrogen and progesterone production in premenopausal women (Fornier *et al*, 2005).

A significant association between C1236T polymorphism and docetaxel clearance was reported by Bosch *et al* (2006), as patients homozygous for the variant T allele showed lower clearance. In the present series, in accordance with this report, patients homozygous for the T allele presented a lower docetaxel clearance although not reaching statistical significance (see Table 5).

In conclusion, these results show that menopausal status at diagnosis has an impact on the effect of C3435T polymorphism of *ABCB1* gene on the pharmacokinetics of docetaxel in breast cancer patients. This finding raises the question of whether higher doses of docetaxel should be given to 3435CC premenopausal women. To provide further insight into this issue, we are currently analysing whether C3435T polymorphism is involved in the pathologic response of breast cancer patients receiving docetaxel in their neoadjuvant chemotherapy regimen according to their menopausal status.

## Figures and Tables

**Table 1:** Patient characteristics (*n*=86)

	**Mean (range) or *n* (%)**	**Missing data *n***
Age at diagnosis (years)	49 (26–76)	0
*Ethnic group*		0
Caucasian	66 (77%)	
North African and sub-Saharan African	19 (22%)	
Asian	1 (1%)	
*Menopausal status* a		0
Premenopausal	53 (62%)	
Postmenopausal	33 (38%)	
Age at menopause (years)	49 (42–59)	10
Height (cm)	163 (149–177)	0
Weight (kg)	67 (46–137)	0
BMI (kg m^−2^)	25 (18–53)	0
Age at menarche (years)	13 (10–18)	42
Age at full-term pregnancy (years)	26 (15–37)	19
Parity	2 (0–10)	6
Ever hormone replacement therapy use	13 (48%)	6
Current smoker	11 (17%)	22
*Tumour stage (T)*		0
T2 (size >20 mm and ⩽50 mm)	53 (62%)	
T3 (size >50 mm)	25 (29%)	
T4 (inflammatory)	8 (9%)	
*Histologic type of breast cancer*		0
IDC	76 (88%)	
ILC	10 (12%)	

Abbreviations: BMI=body mass index; IDC=invasive ductal carcinoma; ILC=invasive lobular carcinoma.

a Menopausal status refers to the menopausal status at the time of diagnosis. Menopause was defined as amenorrhea for at least 12 months.

**Table 2:** Number of patients for each genotype of *ABCB1* polymorphisms

**Polymorphism**	**Genotype**	**Number (%)**
*T-129C*		
	TT	74 (86)
	TC	11 (13)
	CC	1 (1)
A61G	AA	78 (91)
	AG	8 (9)
	GG	0 (0)
C1236T	CC	35 (41)
	CT	38 (44)
	TT	13 (15)
G2677T/A	GG	43 (50)
	GT	32 (37)
	TT	10 (12)
	TA	1 (1)
C3435T	CC	34 (40)
	CT	39 (45)
	TT	13 (15)
61–1236–2677–3435	AA-CC-GG-CC	24 (28)
61–1236–2677–3435	AA-CT-GT-CT	19 (22)
1236–2677–3435	TT-TT-TT	7 (8)

**Table 3:** Pharmacokinetics of docetaxel according to *ABCB1* polymorphisms for premenopausal patients

**Premenopausal patients (*n*=53)**				
	**AUC (*μ*g** **h** **l**^**–1**^**)**		**Clearance (l** **h**^**–1**^**)**	
**Genotype (*n*)**	**Mean (s.e.)**	P*	**Mean (s.e.)**	P*
*T-129C*				
TT (48)	4251 (673)		50.8 (2.6)	
TC (4)	2779 (330)	0.4	52.8 (5.4)	0.8
CC (1)	3410		58.6	
				
*A61G*				
AA (47)	4119 (685)		51.6 (2.6)	
AG (6)	4163(751)	0.4	47.4 (6.5)	0.6
GG (0)				
				
*C1236T*				
CC (21)	3148 (236)		56.1 (4.0)	
CT (24)	5115 (1316)	0.09	48.2 (3.6)	0.3
TT (8)	3713 (380)		46.6 (3.2)	
				
*G2677T/A*				
GG (26)	3093 (192)		56.9 (3.3)	
GT (20)	5560 (1566)	0.02	45.8 (4.1)	0.05
TT (7)	3849 (410)		44.7 (3.2)	
TA (0)				
GG (26)	3093 (192)	0.005	56.9 (3.3)	0.02
GT and TT and TA (27)	5117 (1166)		46.3 (3.1)	
				
*C3435T*				
CC (21)	2727 (104)		61.9 (3.0)	
CT (25)	5431 (1248)	**0.0002**	42.9 (3.4)	**0.0008**
TT (7)	3647 (455)		47.8 (4.4)	
CC (21)	2727 (104)	**<0.0001**	61.9 (3.0)	**0.0002**
CT and TT (32)	5041 (984)		44.0 (2.8)	
				
*2677–3435*				
GG-CC (21)	2727 (104)	**<0.0001**	61.9 (3.0)	**0.0002**
Others (32)	5041 (984)		44.0 (2.8)	
				
*61–1236–2677–3435*				
AA-CC-GG-CC (15)	2638 (131)	**0.0002**	63.3 (3.9)	**0.002**
Others (38)	4711 (836)		46.3 (2.6)	
				
*61–1236–2677–3435*				
AA-CT-GT-CT (13)	6549 (2377)	0.06	43.5 (5.7)	0.08
Others (40)	3336 (179)		53.6 (2.5)	
				
*1236–2677–3435*				
CT-GT-CT (17)	5995 (1829)	0.02	44.1 (4.6)	0.06
Others (36)	3241 (166)		54.4 (2.6)	
				
*1236–2677–3435*				
TT-TT-TT (4)	4097 (725)	0.4	42.4 (5.3)	0.2
Others (49)	4126 (660)		51.8 (2.5)	

Abbreviations: AUC=plasma concentration-time curve; s.e.=standard error.

^*^Comparisons between groups used the Mann–Whitney test for two groups and the Kruskal–Wallis test for three or more groups. Statistical significance was defined as *P*<0.005 (*P* values in bold when *P*<0.005).

**Table 4:** Pharmacokinetics of docetaxel according to *ABCB1* polymorphisms for postmenopausal patients

**Postmenopausal patients (*n*=33)**				
	**AUC (*μ*g** **h** **l**^**–1**^**)**		**Clearance (l** **h**^**–1**^**)**	
**Genotype (*n*)**	**Mean (s.e.)**	***P*** ^*^	**Mean (s.e.)**	***P*** ^*^
*T-129C*				
TT (26)	4635 (351)	0.8	41.5 (3.4)	0.5
TC (7)	4462 (565)		45.7 (5.9)	
CC (0)				
				
*A61G*				
AA (31)	4588 (317)	0.4	43.0 (3.1)	0.4
AG (2)	4765 (15)		33.6 (0.1)	
GG (0)				
				
*C1236T*				
CC (14)	4733 (511)		42.2 (4.4)	
CT (14)	4675 (461)	0.9	41.8 (4.7)	0.9
TT (5)	4006 (517)		44.8 (8.7)	
				
*G2677T/A*				
GG (17)	4612 (425)		42.0 (3.6)	
GT (12)	4228 (414)	0.4	46.2 (5.9)	0.4
TT (3)	4553 (476)		36.9 (5.0)	
TA (1)	8943		20.1	
GG (17)	4612 (425)	0.8	42.0 (3.6)	0.8
GT and TT and TA (16)	4584 (431)		42.8 (4.8)	
				
*C3435T*				
CC (13)	5075 (481)		36.7 (2.8)	
CT (14)	4279 (492)	0.2	46.9 (4.9)	0.3
TT (6)	4309 (539)		44.3 (9.4)	
CC (13)	5075 (481)	0.1	36.7 (2.8)	0.2
CT and TT (20)	4288 (373)		46.1 (4.3)	
				
*2677–3435*				
GG-CC (12)	5141 (517)	0.1	36.4 (3.0)	0.2
Others (21)	4288 (356)		45.8 (4.1)	
				
*61–1236–2677–3435*				
AA-CC-GG-CC (9)	5507 (645)	0.04	34.7 (3.9)	0.09
Others (24)	4257 (311)		45.3 (3.6)	
				
*61–1236–2677–3435*				
AA-CT-GT-CT (6)	4649 (593)	0.8	41.0 (5.8)	0.9
Others (27)	4587 (344)		42.7 (3.4)	
				
*1236–2677–3435*				
CT-GT-CT (6)	4649 (593)	0.8	41.0 (5.8)	0.9
Others (27)	4587 (344)		42.7 (3.4)	
				
*1236–2677–3435*				
TT-TT-TT (3)	4553 (476)	0.7	36.9 (5.0)	0.6
Others (30)	4603 (326)		43.0 (3.2)	

Abbreviations: AUC=plasma concentration-time curve; s.e.=standard error.

^*^Comparisons between groups used the Mann–Whitney test for two groups and the Kruskal–Wallis test for three or more groups. Statistical significance was defined as *P*<0.005.

**Table 5:** Pharmacokinetics of docetaxel according to *ABCB1* polymorphisms for the overall population

**Overall population (*n*=86)**				
	**AUC (*μ*g** **h** **l**^**–1**^**)**		**Clearance (l** **h**^**–1**^**)**	
**Genotype (*n*)**	**Mean (s.e.)**	P*	**Mean (s.e.)**	P*
*T-129C*				
TT (74)	4386 (452)		47.5 (2.1)	
TC (11)	3850 (447)	0.9	48.3 (4.2)	0.6
CC (1)	3410		58.6	
				
*A61G*				
AA (78)	4305 (431)		48.1 (2.0)	
AG (8)	4314 (558)	0.4	44.0 (5.3)	0.5
GG (0)				
				
*C1236T*				
CC (35)	3782 (278)		50.5 (3.2)	
CT (38)	4953 (842)	0.4	45.8 (2.9)	0.5
TT (13)	3825 (297)		45.9 (3.7)	
				
*G2677T/A*				
GG (43)	3693 (232)		51.0 (2.7)	
GT (32)	5061 (988)	0.1	45.9 (3.3)	0.2
TT (10)	4060 (324)		42.3 (2.8)	
TA (1)	8943		20.1	
GG (43)	3693 (232)	0.06	51.0 (2.7)	0.08
GT and TT and TA (43)	4919 (745)		44.5 (2.6)	
				
*C3435T*				
CC (34)	3625 (275)		52.2 (3.0)	
CT (39)	5018 (817)	0.09	44.4 (2.8)	0.2
TT (13)	3953 (347)		46.2 (4.7)	
CC (34)	3625 (275)	0.03	52.2 (3.0)	0.06
CT and TT (52)	4752 (620)		44.8 (2.4)	
				
*2677–3435*				
GG-CC (33)	3605 (283)	0.02	52.6 (3.1)	0.04
Others (53)	4743 (608)		44.7 (2.3)	
				
*61–1236–2677–3435*				
AA-CC-GG-CC (24)	3714 (380)	0.09	52.6 (4.0)	0.2
Others (62)	4535 (524)		45.9 (2.1)	
				
*61–1236–2677–3435*				
AA-CT-GT-CT (19)	5949 (1628)	0.1	42.7 (4.2)	0.2
Others (67)	3840 (189)		49.2 (2.1)	
				
*1236–2677–3435*				
CT-GT-CT (23)	5644 (1355)	0.1	43.3 (3.7)	0.2
Others (63)	3818 (193)		49.4 (2.2)	
				
*1236–2677–3435*				
TT-TT-TT (7)	4293 (437)	0.3	40.0 (3.6)	0.2
Others (79)	4307 (427)		48.4 (2.0)	

Abbreviations: AUC=plasma concentration-time curve; s.e.=standard error.

^*^Comparisons between groups used the Mann–Whitney test for two groups and the Kruskal–Wallis test for three or more groups. Statistical significance was defined as *P*<0.005.
